# A domain-based framework for cognitive profile identification in Parkinson’s disease across diverse samples

**DOI:** 10.3389/fnana.2025.1566835

**Published:** 2025-06-19

**Authors:** Elvira Andújar-Castillo, Carla Carrillo-Molina, Fernando Alonso, Clara Villanueva-Iza, Gustavo Fernández-Pajarín, Angel Sesar, Isabel Jiménez-Martín, Juan Francisco Martín-Rodriguez, Maria Jesús Lama, Pablo Mir, Elena Perez-Hernandez, Cristina Nombela Otero

**Affiliations:** ^1^Departamento de Psicología Biológica y de la Salud, Universidad Autónoma de Madrid, Madrid, Spain; ^2^HM CINAC (Centro Integral de Neurociencias Abarca Campal), Hospital Universitario HM Puerta del Sur, HM Hospitales, Madrid, Spain; ^3^Departamento de neurología, Instituto de Investigación Sanitaria del Hospital Clínico San Carlos (IdISSC), Hospital Clínico San Carlos, Madrid, Spain; ^4^Unidad de Trastornos del Movimiento, Servicio de Neurología, Hospital Clínico Universitario de Santiago de Compostela, Santiago de Compostela, Spain; ^5^Departamento de Neurología, Complejo Universitario de Santiago de Compostela, Santiago de Compostela, Spain; ^6^Unidad de Trastornos del Movimiento, Servicio de Neurología, Instituto de Biomedicina de Sevilla, Hospital Universitario Virgen del Rocío/Universidad de Sevilla/CSIC/CIBERNED, Seville, Spain; ^7^Centro de Investigación Biomédica en Red sobre Enfermedades Neurodegenerativas (CIBERNED), Instituto de Salud Carlos III, Madrid, Spain; ^8^Departamento de Psicología Experimental, Facultad de Psicología, Universidad de Sevilla, Seville, Spain; ^9^Departamento de Medicina, Facultad de Medicina, Universidad de Sevilla, Seville, Spain; ^10^Departamento de Psicología Educativa y Evolutiva. Universidad Autónoma de Madrid, Madrid, Spain

**Keywords:** Parkinson’s disease, cognitive domains, neuropsychological assessment, latent cluster analysis, aging, deep brain stimulation

## Abstract

**Introduction:**

Parkinson’s disease (PD) is a progressive neurodegenerative disorder characterized by motor symptoms and heterogeneous cognitive impairments influenced by factors such as age, disease duration, and severity. Traditional neuropsychological assessments often fall short in capturing the multifaceted nature of PD-related cognitive dysfunction due to their reliance on single test metrics. This study provides empirical support for the implementation of domain-based cognitive assessments, structured in line with Movement Disorder Society recommendations, to provide a multidimensional evaluation of cognitive profiles in PD patients.

**Methods:**

Neuropsychological and clinical data were analyzed from 316 PD patients recruited from three Spanish hospitals—Hospital Clínico San Carlos (Madrid), the University Complejo Universitario de Santiago de Compostela (Galicia), and Hospital Virgen del Rocío (Sevilla)— and a control group of 96 older individuals, whose age difference from the PD group was statistically significant. Five cognitive domains were constructed, addressing attention/working memory, executive functions, memory, visuospatial abilities, and language, using composite z-scores derived from standardized neuropsychological tests.

**Results:**

Latent Cluster Analysis identified three distinct cognitive profiles: (1) a fronto-striatal profile characterized by mild deficits in executive and attention functions and intact visuospatial abilities, (2) a posterior cortical profile marked by severe memory and visuospatial impairments but strong language performance, and (3) a preserved profile displaying mild deficits across multiple domains. Comparisons between PD clusters and controls revealed significant differences in cognitive trajectories, emphasizing the value of a domain-based approach for differentiating neurodegenerative patterns from normal aging.

**Discussion:**

The findings highlight the potential of domain-based assessments to unify data across diverse samples, fostering standardized cross-cohort comparisons and facilitating large-scale research initiatives. By enabling methodological consistency, this approach provides a robust framework for advancing the understanding of cognitive dysfunctions in PD and improving clinical decision-making.

## 1 Introduction

Parkinson’s disease (PD) is a progressive neurodegenerative disorder primarily characterized by motor symptoms such as bradykinesia, rigidity, and tremor, resulting from the degeneration of dopaminergic neurons in the substantia nigra (Fahn and Sulzer, 2004; Parkinson, 1817). However, the impact of PD extends far beyond motor manifestations, encompassing a range of cognitive deficits and non-motor symptoms that profoundly reduce patients’ quality of life (Lang and Lozano, 1998). Among these, cognitive impairments are particularly significant as they can accelerate disease progression and negatively affect independent functioning (Kalaba and Güzeloğlu, 2024).

Mild cognitive impairment in PD (PD-MCI) affects an estimated 20%–50% of patients, and its prevalence increases with disease progression. PD-MCI frequently serves as a precursor to PD dementia (PDD), which affects up to 80% of patients after two decades (Baiano et al., 2020; Greenland et al., 2019; Hely et al., 2008; Litvan et al., 2012). Understanding the cognitive trajectories of PD patients is essential to providing tailored treatments and interventions, particularly as these trajectories are influenced by factors such as older age, longer disease duration and more severe motor complications (Muslimović et al., 2005; Puig-Davi et al., 2024). Consequently, the neuropsychological assessment of PD has become a cornerstone in clinical practice and research.

Despite their utility, neuropsychological assessments often rely on single test scores, which oversimplify the integrated and dynamic nature of cognition. Widely used tools like the Montreal Cognitive Assessment (MoCA) and the Parkinson Neuropsychometric Dementia Assessment (PANDA) demonstrate variability in both sensitivity and specificity. As a result, they often do not fully encompass the range of cognitive impairments, despite being commonly employed as screening tools (Hendershott et al., 2017; Pignatti et al., 2014). This limitation complicates clinical decision-making, particularly in selecting candidates for deep brain stimulation (DBS), the most effective treatment for motor complications but associated with poorer outcomes in cognitively impaired patients. Hence, identifying methods to measure cognitive domains as a whole might be essential for patient evaluation and sample comparisons.

To address these limitations, the Movement Disorder Society (MDS) Task Force proposed diagnostic criteria for PD-MCI, emphasizing a domain-based evaluation approach. These criteria involve two levels: a simplified screening (Level I) and a detailed neuropsychological evaluation (Level II), focusing on domains such as memory, attention, executive functions, language, and visuospatial skills (Goldman et al., 2014; Litvan et al., 2012). Such domain-based assessments better reflect PD-MCI’s heterogeneity, aligning with the underlying neurobiological mechanisms (Halliday et al., 2014). Moreover, they facilitate targeted interventions and improve consistency across clinical studies.

Aggregated domain-specific evaluations provide robust diagnostic insights compared to isolated test metrics. For instance, assessments emphasizing memory and executive functions offer greater diagnostic clarity for PD-MCI (Goldman et al., 2013). However, these benefits are contingent upon the selection of appropriate tools and test combinations. Previous research has demonstrated that the sensitivity and specificity of MDS Level II criteria are influenced by the number and type of tests used (Holden et al., 2014). This underscores the need for methodological rigor when constructing and assessing cognitive profiles. Furthermore, it is crucial to highlight the need to refine existing assessment tools, not only to identify patients with MCI, but also to generate profiles that can help predict the risk of developing dementia. This approach could facilitate more personalized therapeutic decisions.

This study aims to evaluate the validity of constructing cognitive domains through the analysis of neuropsychological and clinical data collected from three specialized centers in Spain. The focus is on patients who are candidates for DBS and a control group significantly older than the patients, enabling the distinction between cognitive changes attributable to normal aging and those resulting from the neurodegenerative mechanisms unique to PD. Comparisons with age-matched controls often underestimate the cognitive impact of PD, since normal aging does not encompass the neurodegenerative processes characteristic of PD (Garagnani et al., 2013). To address this, the study employs domain-based formulas structured around five cognitive domains in PD patients and controls, providing a more accurate framework for understanding PD-specific cognitive impairments.

## 2 Materials and methods

### 2.1 Participants

Clinical data from PD patients were collected from three hospitals in Spain: Hospital Clínico San Carlos de Madrid (*n* = 55; mean age = 56.92 years; σ = 1.35; M/F = 39/16), the Complejo Universitario de Santiago de Compostela (*n* = 188; mean age = 59.98 years; σ = 0.57; M/F = 99/89), and Hospital Virgen del Rocío de Sevilla (*n* = 68; mean age = 55.32 years; σ = 1.09; M/F = 43/25).

Patients included in the study had a confirmed diagnosis of idiopathic PD according to the United Kingdom Parkinson’s Disease Society Brain Bank criteria (Hughes et al., 1992) by a neurologist expert in movement disorders. All participants were candidates for DBS surgery between 2010 and 2022, which necessitated a thorough neuropsychological evaluation. Inclusion and exclusion criteria were applied according to standardized guidelines (Fox et al., 2018). Informed consent was obtained from each participant, adhering to the ethical standards of their respective institutions.

Motor symptoms were evaluated using the Unified Parkinson’s Disease Rating Scale (UPDRS) with medication (on meds) and without medication (off meds) (Fahn and Sulzer, 2004; Goetz et al., 2008). For the aims of this study, just UPDRS III off meds (motor symptoms) will be considered. Additionally, the Levodopa Equivalent Daily Dose (LEDD) was calculated for each participant to quantify medication exposure and assess its potential impact on cognitive functioning (Cervantes-Arriaga et al., 2009; Tomlinson et al., 2010).

Additionally, a control sample (*n* = 96; M/F = 20/76 Age mean = 75.48; σ = 5,52) was included, with data obtained from participants recruited at Seniors Service of the Alcobendas City Council and “Los Nogales” Health Care Centre (both in Madrid, Spain). This sample was characterized by the following criteria: (a) age over 65 years, and (b) no diagnosis of any psychiatric or neurological disorders (c) no cognitive impairment. As for patients, informed consent was obtained from each participant, adhering to the ethical standards of their respective institutions. Descriptives for each sample are shown in Table 1.

**TABLE 1:** Clinical characteristics of the samples.

Clinicals	Samples			
	Santiago	Madrid	Sevilla	Control
*N*	188	55	68	96
Gender (M/F)	99/89	39/16	43/25	20/76
AGE (years)	59.984 (7.799)	56.923 (9.980)	55.324 (9.030)	75.48 (5.52)
LEDD	1323,862 (498.210)	1045.658 (1084.143)	1374.014 (589.644)	–
PD evolution (years)	10.447 (4.111)	11.876 (5.172)	13.319 (5.331)	–
UPDRS III off meds	39.094 (10,752)	35.324 (10.599)	45.131 (10.631)	–

Media values are shown, with the standard deviation in parenthesis.

### 2.2 Neuropsychological assessment

Participants underwent a comprehensive neuropsychological evaluation to assess a range of cognitive functions. Evaluations were conducted by trained neuropsychologists at each hospital, with some variability in protocols across the three institutions. However, all assessment procedures adhered to the MDS recommendations (Litvan et al., 2012). The specific protocols used and the corresponding cognitive domains are detailed in Table 2.

**TABLE 2:** Overview of the neuropsychological assessment of each hospital.

Cognitive Domains	Samples			
	Santiago	Madrid	Sevilla	Control
Attention/WM	DRS Attention subtest^1^ Letters and Numbers (WAIS-III)^2^	DRS Attention subtest^1^ Letters and Numbers (WAIS-III)^2^ TMT A^3^ Direct Digits Span (WAIS-IV)^4^	Letters and Numbers (WAIS-III)^2^ TMT A^3^ Direct Digits Span (WAIS-IV) ^4^ Stroop Part A^5^	TMT A^3^ Direct Digits Span (WAIS-IV)^4^ 5 Digit Test Part 1 and 2 (seconds)^6^
Memory	Immediate Recall of ROCF ^3^ Delayed Recall of ROCF^3^ DRS Memory subtest^1^	DRS Memory subtest^1^ Immediate Recall of ROCF^7^ Differed Recall of ROCF^7^	BVRT^8^	Paired Words (WMS)^9^
Executive Functions	DRS Initiation-Perseveration Subtest^1^ DRS Conceptualization subtest^1^ Phonologic Fluency^10^	DRS Initiation-Perseveration subtest^1^ DRS Conceptualization subtest^1^ TMT B^3^ Reverse Digits Span (WAIS-IV)^4^ Stroop Interference Index ^5^ Phonologic Fluency^10^	TMT B^3^ Reverse Digits Span (WAIS-IV)^4^ Stroop Part C^5^ WCST (Perseverative and non-perseverative errors)^11^	TMT B^3^ Reverse Digits Span (WAIS-IV)^4^ Phonologic Fluency^10^
Visuospatial Function	DRS Construction subtest^1^ Copy of ROCF (time)^7^ JOL^12^	DRS Construction subtest^1^ Copy of ROCF (time and accuracy)^7^ JOL^12^	HVOT^13^	Pentagon Copy^14^
Language	Semantic Fluency^10^	Semantic Fluency^10^ Similarities (WAIS-III)^15^	BNT^16^	Semantic Fluency^10^

References for the test mentioned. Attention/WM:

^1^DRS, Dementia rating scale (Mattis, 1988);

^2^WAIS III, Letters and numbers task (Wechsler and Kaufman, 1999);

^3^TMT, Trail Making Test (Partington and Leiter, 1949), using instructions by Peña-Casanova et al. (2009d);

^4^WAIS IV, Digits subtest Wechsler Adult Intelligence Scale IV (Wechsler, 2008);

^5^Stroop Test (Stroop, 1992);

^6^5 Digit test (Sedó, 2004); ^1,4^Memory;

^7^ROCF, Rey Osterrieth Complex Figure (Rey, 1941);

^8^BVRT, Benton Visual Retention Test (Benton, 1945);

^9^WAIS IVPaired Words task (Wechsler, 2008); ^1,3,4,5^Executive Functions;

^10^Semantic an Phonemic fluency (Ruff et al., 1997), using instructions by Peña-Casanova et al. (2009c);

^11^WCST, Wisconsin Card Sorting Test (Berg, 1948); ^1,7^*Visuospatial function*;

^12^JOL, Benton Judgement of line orientation (Benton, 1994);

^13^HVOT, Hooper Visual Organization Test (Hooper, 1983);

^14^Pentagon Copy (Caffarra et al., 2013) *Language*: ^10,15^WAIS-III, Similarities (Wechsler and Kaufman, 1999);

^16^BNT, Boston Naming Test (Kaplan et al., 1983); *Global Orientation*: MMSE, Minimental State Evaluation (Folstein et al., 1975).

## 3 Statistical analysis

### 3.1 Missing values imputation

A retrospective analysis was conducted on neuropsychological and clinical scores collected from three PD patient cohorts. Analyses were performed using SPSS version 28.0.1.1 (IBM^®^). To address missing data in the dataset, a systematic strategy was employed to minimize potential biases while maintaining data integrity for downstream analyses. Variables with more than 10% missing values were carefully assessed to ensure their exclusion or imputation did not compromise analytical validity.

Missing values were imputed using a machine learning-based predictive approach. Specifically, a Random Forest Regressor was applied to dependent variables, leveraging its ability to model non-linear relationships and handle mixed data types. The regressor was trained on complete rows and then used to predict missing values for incomplete entries (Hong and Lynn, 2020). This hybrid methodology minimized biases and provided a robust dataset suitable for reliable research outcomes (Stekhoven and Bühlmann, 2012).

### 3.2 Comparison across samples

The homoscedasticity of clinical and neuropsychological variables in PD and control samples was assessed using the Kolmogorov-Smirnov test. The results confirmed that the variables did not follow a normal distribution. Detailed results of this analysis are presented in Supplementary Table 1.

To evaluate potential differences in clinical variables among the three PD cohorts (Madrid, Santiago, and Sevilla), a non-parametric Kruskal-Wallis test was employed. This test is particularly suited for independent samples when normality assumptions are violated.

### 3.3 Cognitive domains calculation

Five cognitive domains were defined in accordance with the recommendations of the MDS Task Force (Dubois et al., 2007) and related publications (Goldman et al., 2013) for assessing MCI. Each domain score was constructed as a composite of tests corresponding to that domain. The same methodology was applied to the control group.

Initially, z-scores were computed for each test. For inverse variables—such as those based on error counts or time parameters—adjusted scores (X_adjusted) were calculated using the formula X_adjusted = −X. Z-scores were computed using the formula *z* = (X−μ)/σ, where X represents the raw score (or X_adjusted for inverse variables), μ the mean and δ the standard deviation of the Spanish normative datasets (Campo and Morales, 2003; Lyness et al., 2006; Merten, 2005; Peña-Casanova et al., 2009a,e, b, c, d; Pérez-Enríquez et al., 2021 Wechsler, 2008; Wechsler and Kaufman, 1999).

Subsequently, tests were grouped into cognitive domains as defined in Table 2. Composite domain z-scores were calculated as the mean of the z-scores for all tests within the domain: z_domain = Σz_test/N_tests, where represents the z-score for each test, and is the number of tests in the domain. To facilitate comparisons, composite scores were normalized to a standard range of −3 to 3 using the formula:


z_normalized=[(z_domain-z_min)/(z_max-z_min)]×(3-(-3))-3(3-(-3))-3

To evaluate the internal structure and reliability of the cognitive domains, we calculated Cronbach’s alpha coefficients to assess internal consistency for each domain and site. In addition, we conducted Principal Component Analyses (PCA) separately within each clinical cohort, based on the neuropsychological tests contributing to each domain. Full analytical details and results are reported in the section “4. Results” and Supplementary Table 2.

### 3.4 Data Harmonization in PD samples

The harmonization process for cognitive domain variables followed the guidelines of the Uniform Data Set (UDS-3; Weintraub et al., 2018) to ensure data consistency, completeness, and cross-group comparability. Descriptive statistics were calculated for five cognitive domains — Attention/Working Memory (WM), Executive Functions, Memory, Visuospatial Functions, and Language — to examine central tendency, variability, and the range of scores. The dataset was then stratified by age (40–49, 50–59 and 60–69) and sex (male and female) to assess participant distribution across these demographic groups. Finally, the sample size per age-sex cell was examined to ensure that each cell met the minimum requirement of 10 participants, as specified by UDS-3 harmonization criteria.

To assess the consistency of cognitive scores across sites, Intra Class Correlation Coefficient (ICC) were calculated, using a two-way mixed-effects model, in which subject effects were treated as random and measurement effects as fixed. A consistency definition (Type C ICC) was applied, excluding between-measure variance from the denominator.

### 3.5 Cognitive profile identification

Latent Cluster Analysis (LCA) was performed using the K-means algorithm to identify distinct cognitive profiles among patients. Following cluster identification, PCA was employed to reduce dimensionality and visualize the clusters (Espay et al., 2017).

To evaluate differences between clusters, one-way Analysis of Variance (ANOVA) was conducted, followed by Tukey’s *post hoc* tests, applying the Tukey HSD correction for multiple comparisons. These analyses provided more nuanced insights into variations across clusters.

**FIGURE 1:**
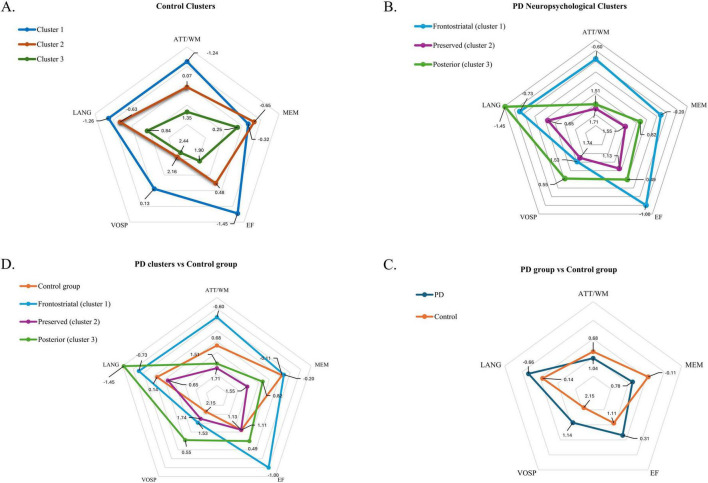
**(A)** Shows a representation of the neuropsychological profiles of the three clusters identified in the control group. **(B)** Represents the profiles of the three neuropsychological clusters identified in the PD group. **(C)** Shows the overall cognitive profile comparison between the control group and the entire PD group. **(D)** Compares the three PD clusters with the control group. The figures display scores across neuropsychological domains: ATT/WM, attention/working memory; MEM, memory; EF, executive functions; VOSP, visuospatial function, and LANG, language. Dots closer to the center of the pentagon indicate higher (i.e., better) cognitive scores, while dots farther from the center represent poorer (more negative) scores.

Subsequently, comparisons between patient clusters and the control group were performed using Independent-Samples Mann-Whitney U tests. Additional clustering analyses within the control sample were conducted for further comparisons (Figure 1A), as detailed in Supplementary Tables 3, 4.

## 4 Results

### 4.1 Descriptive samples analysis

The Kruskal-Wallis test revealed significant differences in age among the three PD cohorts (χ^2^ (2, *N* = 311) = 16.867, *p* < 0.001), years of disease evolution (χ^2^ (2, *N* = 311) = 19.823, *p* < 0.001), UPDRS off meds (χ^2^ (2, *N* = 311) = 24.381, *p* < 0.001) and LEDD (χ^2^ (2, *N* = 311) = 26.232, *p* < 0.001). Pearsons Chi-Square test revealed no significant differences in the sex distribution (χ^2^ (2, *N* = 316) = 8.259, *p* = 0.083).

### 4.2 Data Harmonization in PD samples

Cognitive performance metrics were analysed in the corrected dataset restricted to participants aged 40 and above. Composite scores for Attention/Working Memory (*M* = 1.06, SD = 1.40, range: −3.00 to 3.00) and Language (*M* = −0.67, SD = 1.37, range: −3.00 to 3.00) exhibited moderate variability.

The distribution of participants aligns with clinical criteria for surgical intervention in Parkinson’s disease, which typically targets patients in the first 10–15 years of disease onset. Consequently, age-sex cells in extreme age ranges (40–45 years and > 70 years) naturally have fewer participants, reflecting the rarity of eligible cases in these groups. Despite this, the overall dataset demonstrated sufficient representation across the primary age ranges (50–70 years) and ensured robust statistical power (0.98, medium effect size, α = 0.05) for reliable analyses. These results confirm the harmonization and validity of the dataset for meaningful statistical evaluation, while acknowledging the clinical context of participant recruitment.

Intra Class Correlation Coefficient revealed a low level of concordance across sites. For single measures, the ICC was 0.109 (95% CI: 0.067–0.157), indicating low agreement for individual assessments. For average measures, the ICC increased to 0.380 (95% CI: 0.264–0.483), suggesting fair agreement when considering the average across assessment sites. Both ICCs were statistically significant [F(310, 1240) = 1.614, *p* < 0.001]. These results further underscore the heterogeneity present across cohorts.

### 4.3 Cognitive domains comparison across samples

Table 3 presents the descriptive analysis of neuropsychological composites for each sample. One-way ANOVAs revealed significant differences between PD cohorts in all cognitive domains: attention/WM [F(2,308) = 68.613; *p* < 0.001], executive functions [F(2, 308) = 62.083; *p* < 0.001], memory [F(2,308) = 18.252; *p* < 0.001], visuospatial function [F(2, 308) = 13.224; *p* < 0.001] and language [F(2, 308) = 18.284; *p* < 0.001].

**TABLE 3:** Descriptive analysis of the neuropsychological composite for each sample.

Cognitive Domains	Samples			
	Santiago	Madrid	Sevilla	Control
Attention/WM	1.440 (1.230)	−0.647 (1.199)	1.289 (1.002)	−1.117 (0.363)
Executive functions	0.432 (1.017)	−1.074 (1.134)	1.069 (1.239)	0.166 (0.858)
Memory	1.070 (1.274)	−0.089 (1.087)	0.667 (1.361)	−2.007 (0.346)
Visuospatial	0.874 (1.166)	1.633 (1.125)	1.497 (1.151)	−1.942 (0.589)
Language	−0.904 (1.332)	−0.840 (1.229)	0.181 (1.227)	−1.317 (0.149)

Media values are shown, with the standard deviation in parenthesis.

### 4.4 Psychometric validation of cognitive domains

To assess the reliability and internal structure of the cognitive domains, we performed Cronbach’s alpha analyses and PCA separately for each clinical cohort. In the Madrid sample, the memory and language domains showed acceptable internal consistency (α = 0.72 and 0.79, respectively), whereas lower consistency values were observed in other domains and sites. PCA results revealed 4–5 latent factors per center, explaining 63.6%–71.3% of the total variance, with components generally aligning with the five predefined cognitive domains. Full results are reported in Supplementary Table 2.

### 4.5 Clinical characteristics per PD profile

Latent Cluster Analysis identified three clusters. The elbow method determined that three clusters best represented the data (Na et al., 2010; Collins and Lanza, 2009). Cluster 1 comprised 25.1% of the PD sample (*n* = 78), cluster 2 accounted for 29.3% (*n* = 91), and cluster 3 represented 45.7% (*n* = 142). Details of the clinical characteristics of each cluster are shown in Table 4.

**TABLE 4:** Clinical characteristics per PD profile.

Clinicals	Profiles			
	Cluster 1	Cluster 2	Cluster 3	*p*
AGE	58.600 (0.979)	55.187 (0.981)	60.401 (0.644)	<0.001
Years of PD evolution	12.084 (0.623)	10.819 (0.446)	11.238 (0.381)	0.213
UPDRS III off meds	37.603 (1.219)	40.384 (1.211)	40.518 (0.920)	0.144
LEDD	1160.687 (107.570)	1340.1881 (55.444)	1319.292 (45.158)	0.155

Media values are shown, with the standard deviation in parenthesis.

Analysis of Variance results indicated significant differences among the clusters in age [F(2,308) = 10.606, *p* < 0.001] No differences were found in years of PD evolution [F(2,308) = 1.554, *p* = 0.213], UPDRS III off meds scores [F(2,308) = 1.950, *p* = 0.144] or LEDD scores [F(2,313) = 1.875, *p* = 0.155]. The clinical characteristics of each cluster are detailed in Table 4.

### 4.6 *Post hoc* comparisons and cluster analysis

*Post hoc* comparisons using Tukey’s HSD test revealed significant differences in age across the clusters. Cluster 2 (*M* = 55.19, σ = 0.98) differed significantly from both cluster 1 (*M* = 58.60, σ = 0.98) and cluster 3 (*M* = 60.40, σ = 0.64). However, no significant age difference was observed between cluster 1 and cluster 3. A detailed summary of these comparisons is available in Supplementary Table 4.

### 4.7 Neuropsychological characteristics per PD profile

One-way ANOVAs identified significant differences among the three neuropsychological profiles across all domains, each at the *p* < 0.001 level: attention/WM [F(2, 308) = 129.757, *p* < 0.001], executive functions [F(2, 308) = 97.629, *p* < 0.001], memory [F(2, 308) = 47.188, *p* < 0.001], visuospatial function [F(2, 308) = 41.310, *p* < 0.001] and language [F(2, 308) = 115.874, *p* < 0.001]. Tuckey *post hoc* test tables are detailed in Supplementary Table 5.

Patients in cluster 1 demonstrated relatively strong performance in visuospatial functions (*M* = 1.53, σ = 0.13), however, they displayed moderate deficits in memory (*M* = −0.20, σ = 0.13) and language (*M* = −0.73, σ = 0.12) and strong deficits in attention/WM (*M* = −0.60, σ = 0.13) and executive functions (*M* = −1.00, σ = 0.11). These findings align with the fronto-striatal profile described in the Dual Syndrome Hypothesis, characterized by mild deficits in executive functions and attention but robust visuospatial abilities. The neuropsychological characteristics of each cluster are shown (Figure 1B).

Patients in cluster 2 demonstrated, on average, positive performance in attention/WM (*M* = 1.71, σ = 0.11), memory (*M* = 1.55, σ = 0.11), executive functions (*M* = 1.13, σ = 0.11) and visuospatial abilities (*M* = 1.74, σ = 0.9). A small decline in the language scores (*M* = 0.65, σ = 0.12), appeared in this cluster. Overall, these findings show that this cluster includes “preserved” patients, showing no cognitive dysfunction when compared with other PD patients.

Cluster 3 patients were characterized by marked deficits in language (*M* = −1.45, σ = 0.08) and visuospatial abilities (*M* = 0.55, σ = 0.10). Slight deficits appeared also in executive functions (*M* = 0.49, σ = 0.08) and memory (*M* = 0.82, σ = 0.10), although they were less pronounced than the deficits shown in cluster 1. On the contrary, cluster 3 showed a high positive score in attention/WM (*M* = 1.51, σ = 0.09). This cluster clearly aligns with the posterior cortical profile, characterized by predominant deficits in visuospatial and memory abilities that are poorly responsive to dopaminergic treatments.

### 4.8 Comparison between patients and controls

Independent-Samples Mann-Whitney U tests revealed that the control group was significantly older than the patient group [*z* = 14.030, *p* < 0.001]. In terms of cognitive domains, one-way ANOVAs showed significantly higher scores in the control group for attention/WM [F(1, 405) = 5.122; *p* = 0.024], and memory [F(1, 405) = 35.884; *p* < 0.001]. Conversely, PD patients scored higher in the language domain [F(1, 405) = 26.861, *p* = 0.023], executive functions [F(1, 405) = 29.262; *p* < 0.001] and visuospatial scores [F(1, 405) = 55.828, *p* = 0.093]. Figure 1C presents neuropsychological profile comparisons across PD and control samples.

### 4.9 Comparison between PD profiles and controls

When compared to cluster 1 controls demonstrated significant higher scores in attention/WM (*M* = 0.68, σ = 0.11; *p* < 0.001), executive functions (*M* = 1.11, σ = 0.13; *p* < 0.001), visuospatial function (*M* = 2.14, σ = 0.97; *p* < 0.001) and language (*M* = 0.14, σ = 0.12; *p* < 0.001). No significant differences were found for memory (*M* = −0.11, σ = 0.11; *p* = 0.961).

In comparison to cluster 2, controls showed significantly higher scores in visuospatial function (*M* = 2.14, σ = 0.11; *p* = 0.044). Contrarily, they showed significantly lower scores in attention/WM (*M* = 0.68, σ = 0.11; *p* < 0.001) and language (*M* = 0.14, σ = 0.12; *p* = 0.006). No differences were found in executive functions (*M* = 1.11, σ = 0.13; *p* < 1.000).

Significant differences were also shown when comparing cluster 3 with the control sample. Controls showed higher scores in language (*M* = 0.14, σ = 0.12; *p* < 0.001), visuospatial scores (*M* = 2.14, σ = 0.97; *p* < 0.001) and executive functions (*M* = 1.11, σ = 0.13; *p* < 0.001). However, controls showed significantly lower scores in attention/WM (*M* = 0.68, σ = 0.11; *p* < 0.001) and memory (*M* = −0.11, σ = 0.11; *p* < 0.001). Figure 1D presents neuropsychological profile comparisons across PD clusters and control.

## 5 Discussion

This study provides compelling evidence supporting domain-based cognitive assessments in PD. By analyzing neuropsychological and clinical data from a large cohort of 316 DBS candidates to surgery recruited across three Spanish hospitals, and 96 aged controls, we identified three distinct cognitive profiles—fronto-striatal, posterior cortical, and preserved profiles—that align with findings in the existing literature. Including a control group approximately 20 years older than the PD cohort allowed for robust comparisons, effectively isolating neurodegenerative patterns specific to PD from normal age-related cognitive changes (Garagnani et al., 2013). The domain-based framework illuminated the distinct trajectories of brain degeneration in PD, providing valuable insights into the heterogeneity of cognitive dysfunction. Additionally, this approach introduces a scalable methodology capable of integrating data from larger and more diverse clinical samples, paving the way for improved precision and reliability in heterogeneous cohorts.

### 5.1 Neurobiological underpinnings, clinical implications, and comparison with existing literature

The identification of the fronto-striatal, posterior cortical, and preserved profiles in this study reflects well-documented patterns of cognitive and clinical differences in PD. It aligns with the dual syndrome hypothesis proposed by Kehagia et al. (2013). This framework differentiates between early executive dysfunctions, related to frontostriatal dopaminergic depletion, and more advanced visuospatial and memory impairments associated with posterior cortical atrophy and non-dopaminergic mechanisms. Our findings support this hypothesis while providing additional insights into how clinical and neuropsychological profiles are shaped by disease duration, motor symptom severity, and neurobiological mechanisms.

The preserved profile, characterized by younger patients with shorter disease durations, reflects a more diffuse neurodegenerative process involving both cortical and subcortical regions. Patients in this group exhibited moderate impairments across multiple cognitive domains. This profile aligns with prior studies suggesting that the convergence of dopaminergic depletion and cortical pathology contributes to a broader spectrum of cognitive deficits as the disease progresses (Sampedro et al., 2019). Importantly, the absence of significant differences in LEDD scores across clusters indicates that medication alone cannot explain the observed cognitive heterogeneity, highlighting the interplay of intrinsic neurodegenerative processes. The relative preservation of cognitive function in this subgroup highlights the need to account for specific protective factors when characterizing PD trajectories. Higher cognitive reserve—reflected by greater years of education and regular engagement in mentally stimulating activities—has been linked to delayed onset and milder severity of neuropsychological deficits (Gonzalez-Latapi et al., 2021). In addition, consistent aerobic exercise and resistance training promote neuroplasticity and slow cognitive decline, while adherence to a Mediterranean-style diet rich in antioxidants and omega-3 fatty acids confers further neuroprotective effects (Gu and Xu, 2022). Together, these factors likely interact to shape individual patterns of disease progression in Parkinson’s disease.

In contrast, the posterior cortical profile represents a later stage of PD progression, with significantly older patients. The cognitive deficits in this group, primarily affecting language and visuospatial domains, reflect cortical atrophy in parieto-occipital and medial temporal regions. These findings align with studies demonstrating that posterior cortical dysfunction is less responsive to dopaminergic treatments, implicating non-dopaminergic systems such as cholinergic deficits in driving these impairments (Baiano et al., 2020; Halliday et al., 2014). Neuroimaging evidence further corroborates these results, showing widespread cortical thinning and disrupted network connectivity in advanced PD stages (Aracil-Bolaños et al., 2019). Furthermore, Fernández-Pajarín et al. (2022) provide complementary insights, highlighting how advanced motor symptoms, such as axial symptoms in the “on med” state following subthalamic DBS, indicate distinct neurodegenerative patterns. These findings reinforce the idea that different clinical profiles in PD, such as the posterior cortical subtype, reflect the interplay of disease progression, therapeutic response, and underlying distinctive neurobiology.

The fronto-striatal profile highlights the selective vulnerability of corticostriatal pathways to dopaminergic depletion. This early dysfunction manifests as deficits in executive and attentional processes, consistent with previous neuroimaging studies demonstrating disruptions in striatal connectivity during early PD stages (Mak et al., 2015).

Together, these findings contextualize the cognitive profiles within the broader neurobiological and disease progression of PD. The fronto-striatal profile underscores the early impact of dopamine depletion on executive functions, while the posterior cortical profile reflects advanced neurodegeneration characterized by cortical atrophy and non-dopaminergic dysfunction. The preserved profile provides further evidence for the complex, multifactorial progression of PD. By aligning with established literature and integrating new insights, this study reinforces the utility of domain-based assessments for understanding PD diversity and its neurobiological basis. Given the statistical difference in age between the clusters, it is likely that many patients in the “preserved” profile will eventually evolve into the other profiles.

Clinically, these profiles offer a foundation for developing precision diagnostic and prognostic biomarkers. Frontostriatal dysfunction could be monitored using targeted dopaminergic PET imaging (e.g., ^18^F-DOPA) (Burn et al., 1992) alongside CSF assays of synaptic dopamine turnover (LeWitt et al., 2011), whereas posterior cortical atrophy may be assessed via cholinergic PET tracers (e.g., ^18^F-FEOBV) (Saint-Georges et al., 2021) and CSF measures of acetylcholinesterase activity (Ruberg et al., 1986). Moreover, emerging blood-based biomarkers (such as plasma neurofilament light chain and inflammatory cytokine panels) provide additional molecular insights into subtype-specific neurodegeneration (Ng et al., 2020).

Importantly, genetic factors—such as variants in GBA, APOE, SNCA, and WWOX/MAF—have been shown to influence cognitive trajectories in PD (Chang et al., 2024; Pedersen et al., 2021; Szwedo et al., 2022; Yuan et al., 2025). Integrating such genetic profiles into cognitive phenotyping frameworks may improve the identification of individuals at risk for accelerated decline and inform more personalized treatment approaches.

Leveraging these modalities in longitudinal cohorts would refine patient stratification and enable early detection of progression to Parkinson’s disease dementia (Goldman et al., 2013). Subsequently, this precision phenotyping may inform targeted interventions: dopaminergic agonists combined with executive-function rehabilitation for the frontostriatal profile, and cholinesterase inhibitors plus visuospatial training for the posterior cortical profile. Together, these strategies chart a clear translational path from cognitive phenotyping to biomarker-guided clinical trials.

### 5.2 Advantages and future directions

The domain-based framework employed in this study aligns with current methodologies used in other neurodegenerative disorders, such as Alzheimer’s disease, where domain-specific assessments have improved diagnostic accuracy and enabled cross-cohort comparisons (Federico et al., 2017). By integrating neuropsychological and clinical data, domain-based approaches offer a standardized, scalable method for capturing the complexity of cognitive dysfunction in PD. This consistency facilitates large-scale studies, enhances statistical power, and enables the identification of subtle cognitive patterns that may otherwise remain undetected.

Importantly, this framework also provides a conceptual foundation for the development of next-generation assessment tools that extend beyond cognition to include neuropsychiatric symptoms—such as apathy, depression, and anxiety—as well as measures of social functioning. Recent work on culturally sensitive apathy scales (Yi et al., 2024) integrated cognitive-social instruments (Ya-Wen et al., 2022), and validated social functioning measures in PD (Su et al., 2020) highlight the clinical value of such multidimensional tools. Incorporating these domains may yield a more holistic characterization of disease heterogeneity and facilitate personalized interventions.

Thus, we envision that the present domain-based structure can serve as a foundation not only for cognitive profiling but also for guiding the development of comprehensive, biologically informed clinical instruments.

Despite its strengths, our approach faces several limitations. First, variability in neuropsychological protocols across centers necessitates conversion of raw scores into z-scores, which may attenuate sensitivity to subtle cognitive changes. This heterogeneity is further confirmed by our ICC analysis, which showed low inter-site reliability, indicating inconsistency in assessment procedures across sites. To address this in future research, standardized cognitive batteries —such as the Movement Disorder Society’s recommended assessment toolkit— should be priorized. When harmonization at the acquisition stage is not feasible, statistical harmonization techniques (e.g., ComBat, item-response theory linking) can be employed to align data collected with different instruments.

Second, the construction of cognitive domains from partially overlapping tests across sites may have introduced inconsistency in how each domain was measured. Internal consistency varied across domains and centers, with some domains—such as executive and visuospatial—showing limited coherence, particularly in some sites respect to others. This suggests that the aggregation of heterogeneous tests may have reduced the psychometric reliability of certain domain scores. Additionally, the control group exhibited test heterogeneity, particularly in the visuospatial domain where only the Pentagon Figure test was available, potentially biasing comparisons with patient cohorts. Finally, between 20 and 30% of data were missing in disease progression measures within the two largest subgroups further complicates longitudinal analyses. Incorporating these strategies would enhance the robustness and interpretability of multicentre investigations into PD cognitive trajectories.

While the inclusion of older controls enhances robustness, the PD and control samples were not age matched, and the latter were notably older. Thus, some aging-associated comorbidities may influence the findings when comparing the two groups. Nonetheless, the observed differences suggest that PD follows a distinct trajectory of cognitive decline, different from the one shown in healthy ageing. These findings support the hypothesis that cognitive decline in PD reflects unique pathological processes rather than an accelerated aging pattern, but reflects distinct neurodegenerative trajectories. Future studies should validate these results in longitudinal, multicenter cohorts, incorporating advanced neuroimaging and molecular biomarkers to refine our understanding of PD-related cognitive heterogeneity (Puig-Davi et al., 2024).

## 6 Conclusion

In conclusion, this study provides robust evidence supporting the use of domain-based cognitive assessments to characterize cognitive dysfunction in PD. By identifying distinct frontostriatal, posterior cortical, and mixed cognitive profiles, this study advances our understanding of the neurobiological mechanisms underlying cognitive heterogeneity in PD. These findings align with existing literature and extend current knowledge by linking cognitive phenotypes to disease progression and motor severity. This domain-based framework facilitates both cross-cohort comparisons and the development of tailored clinical interventions aimed at improving diagnostic and prognostic value, paving the way for more targeted and effective management of cognitive dysfunction in PD.

## Data Availability

The raw data supporting the conclusions of this article will be made available by the authors, without undue reservation.
